# Advances in cancer immunotherapy and future directions in personalized medicine

**DOI:** 10.1515/biol-2025-1179

**Published:** 2025-10-29

**Authors:** Yixuan Wang

**Affiliations:** Wuxi School of Medicine, Jiangnan University, Jiangsu, 214000, China

**Keywords:** cancer, immunotherapy, T cells, tumor microenvironment, drug resistance, combination therapy

## Abstract

Cancer immunotherapy has revolutionized oncologic treatment by harnessing and reprogramming the immune system to target malignant cells. This review provides a comprehensive picture of modern approaches to immunotherapy-based treatment strategies, such as immune checkpoint inhibitors (ICIs), chimeric antigen receptor T cells, mRNA vaccines, and biomaterials-based platforms, with a focus on their translational value and application to precision medicine. Emerging insights into the tumor microenvironment, immune resistance mechanisms, and T-cell subpopulation dynamics (e.g., γδ T cells, exhausted CD8⁺ T cells) are analyzed to elucidate immunotherapy response variability. Biomaterials such as injectable scaffolds, nanogels, and artificial antigen-presenting cells enable localized and sustained immune modulation, improving delivery precision and therapeutic efficacy. Personalized approaches, including neoantigen vaccine development and artificial intelligence (AI)-assisted biomarker prediction, are rapidly advancing individualized treatment regimens. Clinical trials illustrate how combination strategies with ICIs, chemotherapy, and nanomedicine enhance patient survival. Despite challenges including immune-related adverse events, manufacturing complexity, and global access disparities, integration of AI and multi-omics platforms promises to optimize patient stratification and therapeutic outcomes. This evolving paradigm positions personalized immunotherapy at the forefront of future oncologic care.

## Graphical abstract

**Figure:**
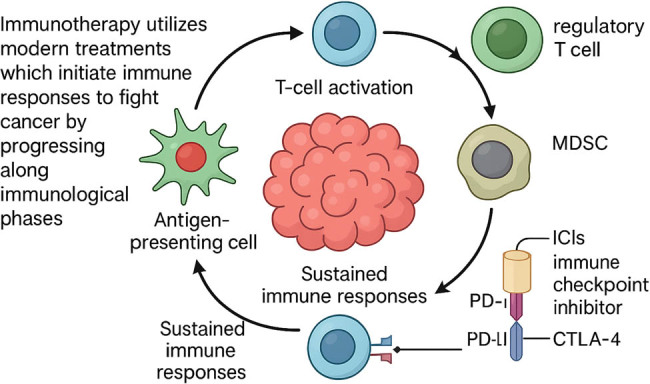

## 1 Introduction

Immunotherapy is a type of cancer treatment that uses the body’s immune system to detect, attack, and eliminate cancer cells. Unlike chemotherapy or radiation, which directly attacks cancer cells, immunotherapy works by boosting the body’s natural immune system to fight the cancer. Cancer immunotherapy (CIT) includes various strategies such as monoclonal antibodies, immune checkpoint inhibitors (ICIs), cancer vaccines, and adoptive cell therapies like chimeric antigen receptor (CAR) T cells, all aimed at counteracting the tumor’s ability to evade immune detection [1,2,3]. The immune system is vital for health because it identifies and destroys defective cells within the body. However, various strategies employed by cancer cells for escape include giving out signals that suppress the immune system or building an environment that suppresses the immune system. Immunotherapy seeks to overcome these drawbacks and, therefore, is a revolutionary treatment method in oncology [4].

One of the most successful immunotherapeutic strategies involves checkpoint inhibition. Molecules like programmed death-1 (PD-1) and cytotoxic T lymphocyte-associated antigen 4 (CTLA-4) normally act as brakes to prevent overactivation of the immune system [5,6]. These inhibitors have proved useful in the treatment of metastatic melanoma, lung cancer, and other cancers. As an example, pembrolizumab and nivolumab have manifested higher survival in patients with previously incurable cancers [7]. CIT is mainly designed to boost the activity of cytotoxic T cells (HLA class I), rather than helper T cells (HLA class II). However, recent studies show that CD4^+^ helper T cells also play a crucial role in fighting tumors [8]. It has functional versatility, which makes these cells develop into subgroups, including Th1, Th2, Th17, and regulatory T cells (Tregs) [9]. On the level of Th1, it stimulates cytotoxic activity and secretion of interferon-gamma to improve anti-tumor effects [10], while Tregs may diminish immune response and form an immunosuppressive environment within the tumor [11]. This bivalency situates CD4^+^ T cells as an attractive therapeutic avenue in next-generation immunotherapies [12].

Another strength of immunotherapy lies in its selectivity – it typically targets cancerous cells while sparing healthy tissues. Targeted therapies include endocrine therapy, molecular targeted therapy, and monoclonal antibodies like trastuzumab for human epidermal growth factor receptor 2 (HER2) positive breast cancer [13,14]. Likewise, bispecific antibodies bind to cancer cells and immune effector cells, enhancing immunosuppressive activity against tumor [15,16,17]. Another factor that makes immunotherapy a potential therapy for cancer and other diseases is its synergistic potential with other therapies. The use of immunotherapy in combination with either chemotherapy [18,19] or radiation boosts the general effectiveness as cancer is treated through multiple approaches. For instance, the integration of radiation therapy with immunotherapy enhances the destruction of tumor cells and diminishes the immune system’s ability to shield the tumor from destruction [20,21]. Furthermore, research has surveyed approaches for improving the concordance of these pairs in order to boost the efficacy of the interventions [22,23]. These combination strategies have been found to prevent the failure of single-agent therapies and enhance the survival of patients with recalcitrant cancers [24].

Among emerging modalities, CAR T-cell therapy involves genetically engineering a patient’s T cells to target specific cancer antigens and has shown remarkable efficacy in hematologic malignancies like leukemia and lymphoma [25,26]. In the same way, oncolytic anti-tumor selective viruses are being genetically developed to destroy cancer cells and trigger immunity [27,28,29]. New investigations also point to the synergism of CAR T therapy and oncolytic virotherapy to enhance efficient treatment strategies [30,31].

To improve efficacy and reduce systemic toxicity, localized delivery of immunotherapies is gaining attention. Interventional radiology allows the local administration of immunomodulators to malignant neoplasms. They have given encouraging outcomes in early trials and are expected in carcinomas that are unresponsive to systemic treatments [32,33,34]. Advancements like hydrogel delivery systems and implantable scaffolds supplement the idea of localized immunotherapy by providing long-term release and action [35,36]. These techniques are growing progressively to overcome impediments resulting from resistant tumors, offering new avenues to curing the disease [37,38].

Immunotherapy, on the other hand, has been more successful in recent years despite certain difficulties. Challenges like immune-related side effects, drug resistance, as well as high costs are still major [39,40]. Also, deciding on the optimal dose and searching for biomarkers that would indicate patient response are two areas of interest for the treatment method. Nevertheless, all those complications make the future of immunotherapy look rather bright. Due to the continuous scientific activities, new ways and techniques will be developed, such as personalized immunotherapy and other new cellular therapies, and global patients’ prognosis will be better and have an increased number of treatments. The change that immunotherapy has brought means that this type of treatment will be central to cancer treatment in the future.

Given the rapid evolution of immunotherapy and its transformative impact on cancer treatment, there is a need to consolidate current knowledge and identify future directions. This review presents both established and emerging immunotherapy strategies – including ICIs, CAR T-cells, and mRNA-based cancer vaccines – while also addressing delivery platforms, artificial intelligence (AI)-assisted modeling, and resistance in the tumor microenvironment (TME). Emphasis is placed on clinical translation and predictive approaches to ensure immunotherapy remains central to modern oncology.

## 2 Overview of the TME

### 2.1 Immune cycle and its four stages in tumor immunity

Immunotherapy is a therapeutic approach to intervene in the body’s immune system with the help of modulators or drugs, which mainly stimulate the body’s immune response to kill cancer cells during the treatment process [41]. The immune cycle in tumor immunity can be broadly divided into four interconnected stages: pre-phase (early stage), mid-phase, mid-late phase, and late phase. These stages collectively describe the process by which the immune system identifies, targets, and eliminates cancer cells. Each phase has distinct events that contribute to the overall anti-tumor immune response, forming the basis for many modern immunotherapies.

During the pre-phase (early phase), tumor antigens are shed off by cancer cells due to either apoptosis or necrosis. These antigen presenting cells (APCs), namely, the dendritic cells (DCs), which then digest them and present them by the Major Histocompatibility Complex (MHC) on the surface of the naive T cells, despite the fact that a T-cell receptor (TCR) with an antigen-MHC, special signals are needed for the activation of the T-cells. This is the foundational step targeted by cancer vaccines and ICIs to enhance T-cell priming and antigen recognition [42]. The mid-phase involves the trafficking of activated T cells toward the TME. This is facilitated under the direction of chemokine gradients, for instance, in CXCL9 and CXCL10. When in the TME, T cells lodge in the endothelium and bind to the cancer cell and recognize tumor antigens presented on the latter through MHC molecules. The engagement of TCRs on T cells with neoantigenic complexes guarantees the selective destruction of cancerous cells. Cytotoxic T cells then induce the apoptosis of cancer cells through such actions as granzyme and perforin-mediated activities. This stage emphasizes the need to traffic T-cells into the tumor; this aspect is helped by some immunotherapies such as ICIs (anti-PD-1, anti-CTLA-4), engineered CAR T cell therapies expressing CXCR3, VEGF inhibitors that normalize tumor vasculature, and chemokine-modulating therapies like nanomaterials enhancing CXCL9 expression. These approaches have been shown to improve T-cell infiltration and enhance anti-tumor immunity [43,44].

**Figure 1:**
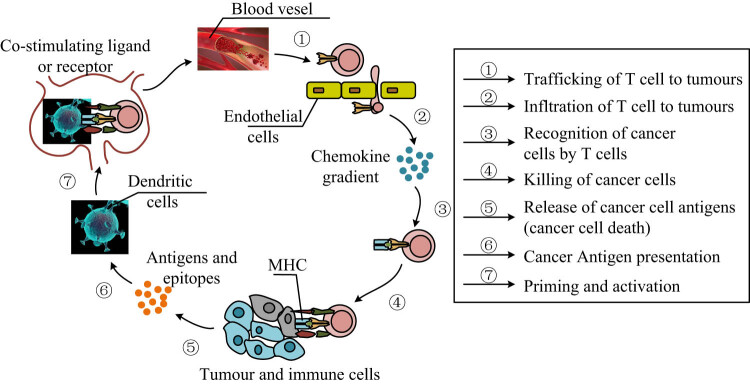
Cancer-immunity cycle depicting the essential steps through which T cells mediate anti-tumor responses. The process begins with the trafficking of T cells to tumor sites via the bloodstream (Step 1), guided by chemokine gradients that facilitate their infiltration into the TME (Step 2). Upon arrival, T cells recognize tumor cells through specific interactions with antigen-MHC complexes (Step 3), leading to the targeted killing of cancer cells (Step 4). This cytotoxic activity results in the release of tumor-associated antigens (Step 5), which are subsequently captured and processed by DCs (Step 6). These APCs prime and activate naive T cells by presenting tumor antigens via MHC molecules and delivering co-stimulatory signals (Step 7), thus initiating a new cycle of immune activation.

During the mid-late phase, the cycle becomes endogenous; that is, the elements that constitute the cycle become continuous and independent. Immune mediated death of tumor cells releases more tumor antigens into the TME as a result of the anti-tumor immune response. Again, these antigens are picked up by the APCs, which boost the immune response by attracting more and more effector T cells. However, this phase is also accompanied by immunosuppressive mechanisms, including the use of Tregs and myeloid derived suppressor cells (MDSCs). In the last period, the immune response against cancer cells is initiated and continued vigorously. Memory T cells generated in earlier stages play a pivotal role in maintaining long-term immune surveillance. These cells maintain the specificity of the primary immune response and are quickly responsive in case of tumor recurrence. Sustained tumor antigen release and continued effector T cell activation result in persistent anti-tumor immunity. Figure 1 displays this cancer immunity cycle and essential steps through which T cells mediate anti-tumor responses.

### 2.2 Molecular mechanisms of immune therapy resistance

**Figure 2:**
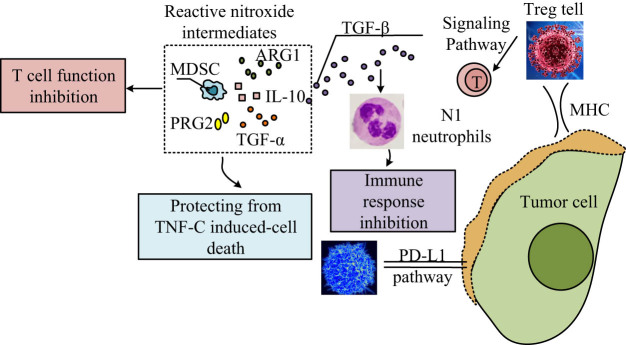
Illustration of the immunosuppressive mechanisms within the TME that contribute to immune evasion and resistance to immunotherapy. MDSCs release a variety of suppressive molecules – including reactive nitroxide intermediates, arginase-1 (ARG1), IL-10, PRG2, TGF-α, and TGF-β – that inhibit T cell function and protect tumor cells from TNF-α-induced cell death. Simultaneously, N1 neutrophils, under the influence of TGF-β and associated signaling pathways, contribute to immune response inhibition. Regulatory T cells (Tregs) further suppress cytotoxic T cell activity by engaging immune checkpoints and altering signaling pathways such as PD-L1, which is often overexpressed on tumor cells to evade immune destruction.

Immunotherapy has revolutionized cancer care by attacking tumors using both cellular and molecular immune responses, and the TME has emerged as an important target of therapeutic response [45]. However, resistance mechanisms, both intrinsic and extrinsic, remain a significant challenge [46]. Tumors can release suppressor cytokines or metabolites and recruit immunosuppressive cells like Tregs and N2 neutrophils. Consequently, treatments such as ICIs also affect cytokine signaling, which can be overcome by tumor-related immune escape [47]. Some of the key resistance mechanisms, as demonstrated in Figure 2, include the MDSC influence on the release of factors such as reactive nitroxide intermediates, ARG1, and interleukin 10, an action which inhibits T-cell activation and prevents the tumor cells from immune mediated cell death [48]. Similarly, Tregs and signaling molecules like TGF-β enhance immune suppression by modulating signaling pathways, including the Programmed death-ligand 1 (PD-L1) pathway, contributing to a highly immunosuppressive TME [49].

Research by Leko and Rosenberg identified different types of tumor antigen recognition mediated by T lymphocytes and explored their clinical safety and efficacy in targeted immunotherapy [50]. Their results imply that enhancing access to antigenic therapy may increase the therapeutic capacity of immunotherapy in cancer patients. Additionally, Zhao et al. demonstrated that activating ferritin decarboxylase could reduce drug resistance in conventional cancer therapy by regulating metabolic pathways and signaling involving iron desiccation [51].

Despite these advancements, resistance remains a significant limitation. For instance, intratumoral heterogeneity and poor immunogenicity in cancers like glioblastoma hinder the success of immunotherapeutic strategies. Murciano-Goroff et al. proposed that overcoming resistance requires both direct tumor modifications and alterations to the TME [52]. Insufficient tumor antigen recognition, ICI modulation, and signaling pathway alterations also play critical roles, as discussed by Bashash et al. [53].

### 2.3 Immunotherapeutic strategies under different treatments

#### 2.3.1 Biomaterials based immunotherapy

Biomaterials based immunotherapy entails the association of fabricated materials aimed at boosting the immune system of the body against diseases, especially cancer. This approach is expected to enhance the effectiveness, selectivity, and safety of immunotherapies by employing biomaterials to deliver and release immunotherapy cargoes and to regulate immune responses [54,55,56].

#### 2.3.2 Implantable and injectable scaffolds for immunotherapy

Implantable and injectable biomaterial scaffolds provide favorable environment on a micro scale for the activation and proliferation of immune cells [57]. For instance, implantable scaffolds have been used to introduce DC-activating agents, making it possible to activate immune reactions *in situ* [58]. Furthermore, these scaffolds may be used for the controlled release of immunomodulatory agents *per se* that engage and stimulate immune cells onsite inside tumors. These implantable biomaterials also have specific release characteristics or kinetic profiles in order to sustain the therapeutic impact. New developments have investigated the incorporation of 3D printing and biotic materials in order to improve the structural and functional properties of such support structures [59].

Injectable scaffolds provide a more minimally invasive method of delivering immunotherapy. These can be prepared as hydrogels or microparticles and can be directly implanted into the desired site for drug delivery, where the system, upon implantation, undergoes self-assembly or gelation to form a reservoir for the drug. In particular, injectable biomaterials have been proven to be practical for metastatic cancers and for regions that are difficult to operate on. In this way, they can secrete cytokines or ICIs to remodulate the immune context of tumors [60]. However, the injectable scaffolds can be administered with any concentration and can easily accommodate combination therapy. Research has shown their potential in the delivery of multiple agents, including tumor antigens and adjuvants to boost immunity [56].

Both implantable and injectable scaffolds have a similar benefit of targeted delivery, therefore reducing toxicity associated with systemic exposure. It is possible to design such systems to only respond to certain biological signals, thus enabling the delivery of therapeutic agents to occur based on changes within the microenvironment. Combined application of these scaffolds with other treatment strategies, including ICIs or CAR T cell therapy, is a relatively new trend that has the potential to provide enhanced and individualized immunotherapies [37].

#### 2.3.3 Nanoparticle-based delivery systems

Immunotherapeutic agents are delivered through nanoparticles to target specific immune cells or tumor sites. Liposomes and polymersomes as synthetic nanoparticles can be designed and coated with tumor antigens or immune modulating agents to give high immune response signals. Also, the use of biologically derived nanocarriers such as extracellular vesicles has been considered owing to their inherent biocompatibility and immune-associated functions [61]. Nanoparticles act to encapsulate immunotherapeutic agents, including antigens, adjuvants, or immune modulators, in order to enhance their stability and have slow-release properties. For example, nanoparticulate based vaccination offers more efficient antigen deposition and presentation, with resultant improvement in immune response in cancer immunotherapies [62]. In addition, lipid based nanoparticles, gold nanorods, and mesoporous silica nanoparticles have also been used for enhancing the DC uptake, which is crucial for the activation of the immune system [63].

Furthermore, in recent years, nanocarrier-based biomedicine has made significant progress in cancer therapy. Ma et al. analyzed the research progress of nanogels applied to immunotherapy, including gel-mediated immunomodulatory drugs, antibodies, vaccines, and CAR T cell therapy [64]. This study concluded that nanogels had better application potential for immunotherapy. As a three-dimensional crosslinked aqueous material, nanogels are structurally stable, have a large specific surface area, and can better target particular immune cell subpopulations or immunological organs, which boosts the drug’s bioavailability. Additionally, fewer adverse events are produced by its slower release impact. Zhang and Pu proposed to combine fusion nanomedicines with immunotherapy and discussed tumor immunotherapy under molecular and nanoengineering approaches [65]. This study concluded that activatable nano-formulations could produce immunotherapeutic effects on TME under internal and external stimuli and reduce the incidence of immune-related adverse events. Xu et al. prepared a personalized nano vaccine based on a cationic fluoropolymer and achieved control of melanoma with the help of a Toll-like receptor 4 mediated signaling pathway [66]. The results showed that this method could better inhibit tumor recurrence and metastasis after surgery.

Another notable application involves using nanoparticles to overcome resistance in the TME. As an example, nanomedicines that target tumor-associated macrophages or other immune-suppressive cells have demonstrated efficacy in augmenting therapeutic efficacy in drug-resistant tumors [67]. Moreover, the controlled and local delivery of immune-stimulating agents can be achieved using polymer-derived nano and microparticle carriers [68]. The adaptability of nanoparticle-based systems allows for the integration of multiple therapeutic functionalities. They can administer drugs and immune stimulants either simultaneously or sequentially so as to capitalize on the synergies. As an example, paclitaxel combined with engineered nanoparticles has been utilized as carriers that may also alter immune response as a cancer therapy strategy [69].

#### 2.3.4 Modulation of the TME

Implanted biomaterials can be bioengineered in a way to modulate the TME and encourage immune cell infiltration and effectiveness. For instance, biomaterials that let out oxygen counter tumor hypoxia, which tends to dampen the immune response. Other strategies include delivering agents that are capable of breaking down the surrounding matrix and thus increasing immunological access [70]. Based on their findings, Martin et al. asserted that TME acts as a barrier to the release of drugs and thus negatively affects the effectiveness of nanomedicine and immunotherapy, which may lead to increased drug toxicity and adverse events in malignant tumors [71].

#### 2.3.5 Artificial antigen presenting cells (aAPCs)

aAPCs are artificial systems designed to resemble the natural APCs to activate T cells *ex vivo* or *in vivo*. The construction of aAPCs includes the use of biodegradable materials or nanoparticles, which are functionalized with signals that are critical in the activation of T cells. These include the display of ligands both in MHCs and co-stimulatory molecules that make sure that there is a firm T-cell activation. For example, the use of nanoellipsoidal aAPCs demonstrated much potential for antigen specific T-cell activation due to a large surface area for contact [72]. The effectiveness of aAPCs can also depend on how they have been designed. Some investigations gave evidence that the shape of the particle and its material affect the activation of T-cells at supra-physiological levels. For example, ellipsoidal nanoparticles and lipid based aAPCs hold the biological model of natural APCs to provide signals [73]. Another example is structured cell-sized lipid vesicles with protein coupled bilayer layers showing antigen-specific T-cell stimulation [74].

Furthermore, aAPCs possess antigen presentation versatility for multiple immunotherapeutic uses, such as adoptive cell transfer. By altering these design parameters, including the antigen density and co-stimulatory molecules, they can improve T-cell proliferation and activation prior to the therapeutic delivery. These systems have a particular potential to avoid the drawbacks inherent to natural APCs, such as interferences with immune reactions and scarcity of supply. The possibilities of aAPC are not only limited to identifying the immune targets in CIT but also to overcoming immune issues. Particle-based aAPCs have been developed to overcome the immunosuppressive TME by activating tumor reactive T-cells [75].

#### 2.3.6 Bioinstructive materials

Bioconductive materials are designed for specific cellular responses such as differentiation, proliferation, and migration. For instance, synthetic materials that were produced to specify certain pore sizes were used in works that illustrated that mesenchymal stem cell differentiation is affected by the structure of materials [76]. Likewise, electrospun nanofibers function as scaffolds that can control differentiation through Mechanotransduction pathways in musculoskeletal, vascular, as well as immune systems [77]. Bioinstructive materials also require the ability to influence the immune system response. For example, immune-instructive biomaterials are under development for improving the foreign body response and tissue integration and functioning of implants, maintaining immunological tolerance [78]. These materials apply chemical and physical features to modulate the activities of immune cells so as to initiate or inhibit immune response.

Recent advancements in technology, for instance, High-Throughput Screening technology, help scientists find efficient bioinstructive materials for particular usage. Screening techniques based on gradient profiling have been especially successful in the context of cell-biomaterial interactions and specific control of cell behavior, including proliferation and differentiation [79]. Bioinstructive scaffolds have been applied to develop an environment for therapeutic cell production *in situ*. For instance, implantable scaffolds have been developed to synthesize and deliver CAR T-cells within the body, thereby synchronizing immunity across different types of complex cell therapy techniques [80]. These innovations emphasize the possibilities of bioinstructive materials in improving the outcome of act T therapies. This field is already transitioning to dual-mode applications where implants provide both structural support and deliver bioactive cues in conjunction with other forms of treatment, including drug delivery and gene modification. For example, bioinstructive hydrogels have been designed to create pronto environments for implantation as well as enhanced tissue healing [81]. Table 1 summarizes current innovations in biomaterial-based platforms such as injectable scaffolds and hydrogels, highlighting their composition, mechanism of action, and tumor-specific applications.

**Table 1:** Innovative biomaterial platforms for CIT

Category	Biomaterial type	Example	Tumor type/application	References
Injectable scaffolds	Nano-vaccine in hydrogel (NvIH)	NvIH hydrogel with ICB antibodies and polymeric nanoparticles activating TLR7/8/9 and STING	Glioblastoma and poorly immunogenic tumors	[82]
Injectable scaffolds	L-Norvaline-based hydrogel	Hydrogel blocking ARG1 pathway for T-cell reactivation and combination immunotherapy	Primary tumors, abscopal tumors, pulmonary metastasis	[83]
Injectable scaffolds	PEI-based immunotherapeutic hydrogel (PEIGel)	PEIGel hydrogel with polyvinyl alcohol, polyethylenimine, and magnesium ions to upregulate PD-L1 and M1 macrophages	Breast cancer, tumor relapse prevention	[84]
Injectable scaffolds	Sodium alginate-based	Injectable sodium alginate hydrogel for deep tumor penetration and personalized vaccines	Colorectal cancer	[85]
Injectable scaffolds	Supramolecular hydrogel for *in situ* CAR T reprogramming	Hydrogel loaded with plasmid CAR (pCAR) with CD2 promoter for enhanced CAR T therapy	Solid tumors (Humanized mice models)	[86]
Injectable scaffolds	Silk fibroin (SF) microsphere-based vaccine	Macroporous SF microsphere loaded with antigens and adjuvants for single-dose cancer vaccination	General solid tumors	[87]
Injectable scaffolds	Super-soft DNA hydrogel depot	Injectable DNA hydrogel with ATP aptamers for sequential doxorubicin, CpG ODN, and aPDL1 release	General solid tumors	[88]
Cancer vaccine hydrogels	Nucleic acid-based hydrogel vaccine	Three-in-One hydrogel with CpG, SN38, and PD-L1 siRNA for immune activation	Melanoma (Murine model)	[89]
Cancer vaccine nanocomposites	Oxidized bacterial cellulose/thrombin/gold nanocomposites (AuNCs)	Nanocomposite with αPD-1@AuNCs for pyroptosis-inducing photothermal and ICI therapy	Postoperative head and neck squamous cell carcinoma (HNSCC)	[90]
Cancer vaccine hydrogels	Bio-responsive cargo-catchable gel depot	Pullulan/chitosan hydrogel for doxorubicin and aPDL1 co-delivery to prevent tumor recurrence	Post-surgical breast cancer (4T1 Mouse model)	[91]

### 2.4 Immunotherapy in cancer management

**Figure 3:**
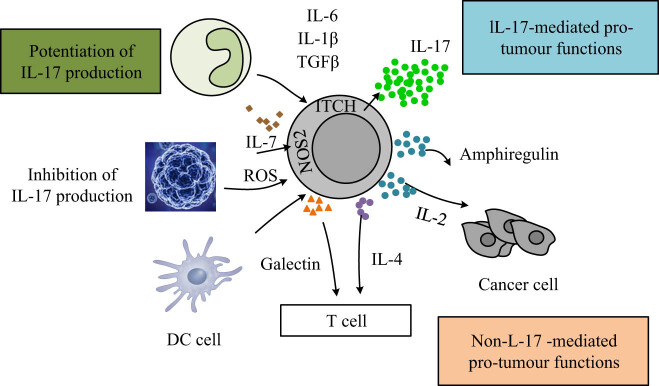
Regulatory mechanism of tumor promoting gamma delta T-C. IL-17 production, driven by ITCH and NOS2 signaling, promotes inflammation and tumor growth. Amphiregulin and IL-4 further enhance cancer cell survival. Conversely, ROS from DCs and galectin signaling can inhibit IL-17 production.

Immunotherapies ranging from ICI blockade to cellular therapeutic vaccines have changed the paradigm of tumor treatment. Among them, T-cell antigen specificity has been shown to have a link to tumor phenotype. The utilization of T-cell subsets is discussed to be important for elucidating the anti-tumor cellular mechanisms of immunotherapy and guiding treatment regimens [92]. T-cell serves as the most potent effector cells in the anti-tumor immune response. In current CIT, T-cells are mainly used to achieve interventions from both ICIs and overt cell therapy. Chow et al. analyzed the immunobiology of T-cell exhaustion and noted its correlation with tumor control. They emphasized that targeting exhaustion pathways could significantly enhance immunotherapy efficacy [93]. T-cell-based immunotherapy has become an effective therapeutic strategy for anti-tumor therapy in addition to surgery, chemotherapy, and radiotherapy. Based on the differences in cell receptor expression, T-cells can be classified into two subpopulations, αβ T and γδ T. Mensurado et al. concluded that current αβ T-C are more dependent on MHC-mediated and neoantigen expression, which limits their applicability. In contrast, γδ T-C were less dependent on neoantigen loading and MHC antigens due to their unique tissue orientation, allowing them to have better anti-tumor activity. The study of γδ T-cell subsets could provide a reference value for cancer prognostic intervention and therapeutic strategy development [94]. Figure 3 shows the regulatory mechanism of tumor-promoting γδ T-C.

Natural Killer (NK) cells resemble a significant part of the innate immune system and can recognize and kill tumor cells. Recent literature has isolated certain NK cell subsets, including RAC1 high NK cells, which exhibit augmented tumor killing abilities in hepatocellular carcinoma (HCC) via regulating the STAT3-NKG2D axis [95]. Single-cell sequencing has advanced the understanding of the tumor immune microenvironment (TiME), revealing distinct immune subpopulations. As an example, T-cell, B-cell, and myeloid secondary clustering have been used to make predictions regarding the responsiveness of melanoma to immunotherapy [96]. These insights enable the development of therapies targeting immune subpopulations critical to anti-tumor immunity. In colorectal cancer, subpopulations of high immune infiltration defined by the peroxisome pathway and TIM3 expression are associated with better immunotherapies. This illustrates the role of understanding individual immune parameters in informing patient-specific therapeutic approaches [97]. Terminally exhausted T-cells, marked by TOX and PD-1 expression, present challenges in solid tumors. Blocking PD-1 and TIGIT in a co-blockade approach has been demonstrated to renew these exhausted cells, increasing their anti-tumor capacity in bladder cancer [98]. DCs are pivotal for initiating adaptive immunity. The heterogeneity of monocyte-derived and type-2 conventional DCs has been shown in single-cell analysis, underscoring their value in custom applications in immunotherapy [99]. Subpopulation-based approaches also address limitations in CAR T-cell therapy. Using omics technologies to analyze the tumor immune microenvironment, scientists are finding ways to boost the effectiveness of CAR T-cells in non-T-cell-inflamed tumors [100]. In glioblastoma, stromal subpopulations, such as perivascular fibroblasts, have been identified as predictors of immunotherapy resistance. It is noteworthy that this observation highlights the significance of addressing both immune cells and stromal subpopulations to maximize treatment effects [101].

A discussion of CAR T-cells vs bispecific antibodies presents varying efficacy, toxicity, and cost profiles (Table 2). CAR T has shown maximum efficiency in hematological malignancies but is fraught with cytokine release syndrome (CRS) and neurotoxicity, excessive costs, and production delays. Moreover, unlike monoclonal antibodies, bispecific antibodies like blinatumomab are enabled in the form of an off-the-shelf product, at lower cost and higher access, especially through a community-based model. However, they generally have lower persistence and moderate efficacy compared to CAR T [102].

**Table 2:** Comparative evaluation of CAR T-cells and bispecific antibodies

Parameter	CAR T-cell therapy	Bispecific antibodies (BsAbs)	References
Efficacy	High response rates in relapsed/refractory hematologic malignancies; durable remissions in Large B-Cell Lymphoma and Multiple Myeloma; Overall Response Rate (ORR) >50% in many trials	Rapid response onset; effective in hematologic cancers; responses less durable compared to CAR-T	[103,104]
Toxicity	High: CRS, neurotoxicity; requires inpatient care and close monitoring	Lower: lower-grade CRS and neurotoxicity; more manageable in outpatient settings	[105,106]
Cost (per patient)	Estimated ∼$375,000–$475,000 (not including hospitalization, supportive care); highest upfront cost	Estimated ∼$180,000–$250,000; generally lower than CAR T but cumulative costs increase with long-term use	[107,108]
Turnaround time	2–3 weeks for cell engineering; logistical complexity due to autologous manufacturing	Off-the-shelf, immediate availability	[109,110]
Ideal use case	Curative intent in fit, relapsed/refractory patients; aggressive or resistant cancers	Bridge to CAR T or alternative for less fit patients, outpatient settings	[106,111]

### 2.5 Immune checkpoint-based immunotherapy

**Figure 4:**
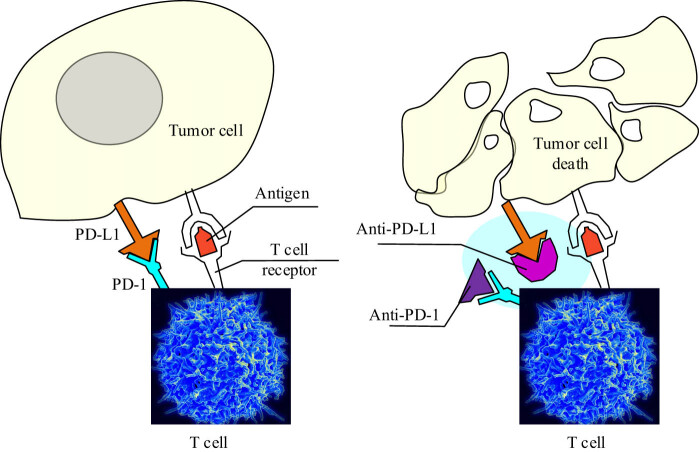
Mechanism of immune checkpoint inhibition targeting the PD-1/PD-L1 axis in CIT. On the left, tumor cells express PD-L1, which binds to PD-1 receptors on activated T cells, leading to suppression of T cell-mediated immune responses and allowing tumor cells to evade immune destruction. On the right, the administration of anti-PD-1 or anti-PD-L1 monoclonal antibodies blocks this interaction, thereby restoring T cell activity. This blockade enables T cells to recognize tumor antigens through the T cell receptor (TCR) and induce tumor cell apoptosis.

Approved in 2011, the first generation of ICIs targets tumor cells by interfering with the inhibitory signals that the tumor cells send to immune effector cells. Its immunostimulatory properties may lead to the occurrence of associated adverse reactions and damage to organ systems. Chhabra and Kennedy concluded that there was a need for greater multidisciplinary coordination in responding to the management of adverse reactions to achieve individualized protocol development for the patient’s therapeutic goals [112]. Figure 4 shows the structure of PD-1 and PD-L1.

**Figure 5:**
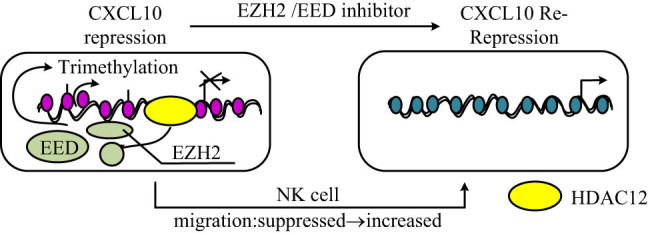
Regulatory mechanism of trimethylation on the HCC TME. The EZH2-EED complex facilitates CXCL10 repression through histone trimethylation, suppressing NK cell migration. Upon treatment with an EZH2/EED inhibitor, CXCL10 expression is restored, leading to increased NK cell migration. However, HDAC12 can induce CXCL10 re-repression, maintaining a suppressive TME.

ICIs like PD-1/PD-L1 and CTLA-4 inhibitors have been particularly effective in improving survival outcomes in several cancers, including HCC, melanoma, and non-small cell lung cancer (NSCLC) [113,114,115]. In HCC, antibody-based ICIs enhance T-cell responses within TME, contributing to improved therapeutic efficacy. Figure 5 shows the role of trimethylation in regulating immune responses within the HCC TME.

In cancer therapy, anti-tumor immune (anti-TI) effects can be enhanced through neoadjuvant ICI blockade methods. Topalian et al. suggested that research on checkpoint blockade responses and drug resistance markers holds potential for further development [116]. Koerner et al. discovered that double-stranded (ds) RNA adjuvants effectively activate DCs and increase tumor-specific CD8^+^ T-cell responses [117]. ICI blockade reactivates cytotoxic T lymphocyte (CTL) responses, and its combination with biodegradable polylactic acid-co-glycolic acid (PLGA) particles significantly improves immunotherapeutic efficacy.

Targeting emerging pathways like the IGSF8–NK receptor axis also offers promise. Disrupting these interactions, alone or with PD-1 antibodies, enhances cytotoxic function and T-cell signaling [118]. Despite their effectiveness, ICIs are associated with irAEs such as inflammation and organ toxicity. This is due to their multifaceted modulation of immune pathways [119]. Sharma et al. noted that resistance to ICIs is often tumor-type dependent, highlighting the importance of identifying predictive biomarkers to guide personalized treatment [120]. In gliomas, O6-methylguanine-DNA methyltransferase (MGMT) status has been associated with ICI resistance. Kinslow et al. reported a negative association between MGMT staining and primary brain tumor risk scores, suggesting its potential as a biomarker for ICI responsiveness in glioma [121]. Monoclonal ICIs prevent T-cell inactivation by blocking ICIs, effectively enhancing antigen recognition and cytotoxicity [122]. However, phenomena like hyperprogression and irAEs remain major barriers to broader application [123]. These therapies do this by rewriting the antigens of the immune system so that they can recognize the tumor.

Originally developed for oncology, ICIs are now being explored for other immune-regulated diseases like autoimmune disorders and atherosclerosis, where checkpoint modulation may regulate inflammation and plaque formation [124]. Recent research is also expanding ICI use beyond T-cells – investigating NK cells and single-cell transcriptomic profiling to reveal how checkpoint pathways affect diverse immune subsets [125,126]. This has helped inform combination therapies with small molecule inhibitors or radiotherapy that may enhance treatment efficacy while minimizing toxicity [127].

### 2.6 Combination immunotherapy

Combination immunotherapy represents a versatile and increasingly prominent strategy in cancer treatment, aiming to overcome limitations associated with single-agent therapies (Table 3). While many nanomedicines are in development, challenges persist – including over-activation of immune responses, increased immunogenicity, and drug resistance [128]. TME modulation strategies are particularly critical for enhancing immunotherapy outcomes. Traditional immunotherapeutic drugs often have a short retention time during administration, limiting their effectiveness. Yang et al. concluded that nanoparticle-based drug delivery systems can effectively penetrate the TME and release drugs with precision. These systems could also be combined with other therapeutic approaches, although careful control of TME changes at different stages remains key to designing personalized nano-application platforms in the future [129].

**Table 3:** Summary of some combination immunotherapy strategies

Strategy	Description	Examples	Benefits	Challenges	References
ICIs in combination	Combining ICIs targeting PD-1/PD-L1 and CTLA-4 pathways for synergistic effects	Nivolumab + Ipilimumab in metastatic melanoma	Enhanced tumor clearance and survival	Increased risk of immune-related adverse events (irAEs)	[130,131]
ICIs and targeted therapy	Combining ICIs with tyrosine kinase inhibitors to modulate the TME	PD-1 inhibitors with VEGF inhibitors in NSCLC	Synergistic immune activation	Managing complex dosing and cumulative toxicities	[132]
ICIs and adoptive cell therapies (ACTs)	Combining checkpoint blockade with CAR T-cells or TCR-engineered T cells	Anti-PD-1 + CAR T-cells for solid tumors	Prevents T cell exhaustion	Complexity of therapy design and delivery	[133]
Dual immune modulators	Targeting multiple immune pathways to enhance anti-tumor responses	IL-2 agonists with ICIs	Activates diverse immune populations	Increased systemic immune activation risks	[134]
ICIs and oncolytic viruses	Using viruses that selectively infect and kill cancer cells, combined with ICIs	T-VEC (Talimogene laherparepvec) with anti-PD-1 therapy	Increases tumor immunogenicity	Optimizing virus delivery and immune responses	[135]
ICIs and chemotherapy	Enhancing antigen presentation and immune activation through combined mechanisms	Anti-PD-L1 + platinum-based chemotherapy in lung cancer	Broader efficacy across cancers	Higher rates of irAEs and systemic toxicity	[136]

PD-1 inhibitors such as pembrolizumab or atezolizumab, when combined with chemotherapy, have emerged as significant therapeutic strategies for non-immunogenic malignancies. This combination has demonstrated better clinical activity and increased the median progression-free survival of patients. However, uncertainties in the pathways activated by immunotherapy in breast cancer complicate its application in advanced triple-negative breast cancer, HER2+ breast cancer, and estrogen receptor-positive breast cancer [137]. Torres and Emens suggested that immunomodulation strategies, including checkpoint inhibition, bispecific antibodies, and drug eurythmy, could activate immune responses more effectively [138].

Single-agent anti-PD-1 ICIs have shown efficacy in early HCC treatment. Later-stage studies incorporated atezolizumab in combination with bevacizumab, which was approved as a first-line treatment modality. This combination significantly improved response and survival rates. Enhancing the combined use of ICIs or combining them with targeted therapies remains an important focus of future clinical research [139]. In advanced or metastatic gastric cancer, ICIs combined with chemotherapy and HER2-targeted treatments have shown promise. For instance, patients with PD-1 positivity exhibited higher overall survival rates when injectable olaparib (nivolumab) was combined with chemotherapy [140].

To overcome challenges associated with CAR T-cell therapy in hematologic malignancies, especially related to antigen heterogeneity and TME induction that limit the cytotoxic effects and the payload, Rezaei et al. suggested a synergistic strategy of a combination of lysosomal viral therapy and immunotherapy. This targeting approach selectively localizes therapeutic agents within the tumor milieu and can be used as a prototype in the treatment of solid malignancies [25]. The clinical advancement and identification of ICIs against CTLA-4, PD-1, and PD-L1 have transformed cancer treatments. Further discussing the modulation of T-cell functions, Kraehenbuehl et al. summarized co-stimulatory and co-inhibitory TCRs. They highlighted that a better optimization of co-inhibitor strategies should focus on tumor- and patient-specific properties. The use of targeted immunotherapy employing poorly co-stimulated receptors, cell subtype-specific targeting, and manipulating tumor cell metabolism may enable personalized treatment regimens [141].

### 2.7 Advancements in personalized CIT

Personalized medicine supports better treatment decisions through analysis of individual patients’ genetic information and tumor abnormalities, and biomarkers to identify optimal therapy options. The main barrier in personalized medicine practice emerges from uniting information from biological data with clinical choices to enhance cancer treatment efficiency [142]. The main advancement in personalized medicine involves targeted therapies and immunotherapies that exclusively target cancer cells yet protect healthy tissue. The use of HER2 inhibitors in breast cancer treatment, alongside EGFR inhibitors in lung cancer treatment, has proven more effective than traditional chemotherapy methods [143].

Unlike conventional “one-size-fits-all” approaches, personalized medicine tailors treatments based on individual patient characteristics, including tumor heterogeneity. Table 4 shows varied comparisons of traditional and personalized cancer treatments with differences in treatment approach, efficacy, and patient specificity highlighted. Research shows that traditional chemotherapy and radiation treatments succeed in specific cancers, but various treatment response rates exist among patients diagnosed with the same cancer type because of tumor heterogeneity [144]. The integration of personalized medicine techniques with conventional cancer treatments aims to maximize therapy results rather than substitute existing treatments. Genomic profiling enables doctors to identify patients who will benefit from chemotherapy, thus helping them avoid giving worthless treatments. A combination of precision medicines together with standard cancer treatments has resulted in promising outcomes. Targeted agents used together with radiation therapy generate stronger tumor control and minimize radiation resistance [145].

**Table 4:** Differences between traditional and personalized cancer treatments

Aspect	Traditional cancer treatment	Personalized cancer therapy
Approach	Standardized for all patients	Tailored to individual molecular profiles
Targeting	Non-specific (affects cancerous and healthy cells)	Tumor-specific antigens and molecular pathways
Side effects	Higher toxicity, off-target effects	Reduced toxicity due to precision targeting
Effectiveness	Variable response across patients	Higher efficacy in selected patient populations
Examples	Chemotherapy, radiation, surgery	CAR T-cell therapy, checkpoint inhibitors, neoantigen vaccines

#### 2.7.1 Neoantigen-based cancer vaccines

Personalized cancer vaccines employ tumor-specific mutant proteins known as neoantigens to induce immune responses that are tailored to each patient. In laboratory studies, neoantigen-based vaccines have proven to enhance T-cell activation and their specificity, leading to better destruction of tumors by immune responses [146]. Recent studies have shown an immense potential of personalized cancer vaccines in clinical practice of treating melanoma and other solid tumors [146]. Studies indicate that there are a maximum of 20 predicted cancer-specific neoantigens in every patient, which makes personalized vaccines a promising method within the framework of targeted immune response [147]. The feasibility of such vaccines and the resulting robust immune response was confirmed through multiple clinical trials, which made them effective tools in precision immunotherapy [148].

#### 2.7.2 Precision oncology and immunotherapy integration

Cancer treatment through precision medicine combined with immunotherapy has become revolutionary, specifically in colorectal cancer management. The identification of biomarkers, together with molecular profiling advances, allows healthcare providers to create personalized treatment plans that increase treatment effectiveness [149]. The future of ICI’s development focuses on both enhancing resistance-breaking abilities and attaining superior therapeutic outcomes [150]. Recent evolutions in CAR T-cell therapy focus on developing multiple tumor antigen recognition systems to prevent tumors from escaping immune responses. Tisagenlecleucel and Axicabtagene-ciloleucel represent the first approved CAR T therapeutic agents that enable research into enhanced cellular immunological treatments [151].

#### 2.7.3 Next-generation biomarkers

Biomarker detection serves as the fundamental basis for enhancing precision medicine applications in CIT. Biomarker-based stratification continues to enhance treatment selection through which clinicians can anticipate patient reactions to ICIs, as well as CAR T therapy and cancer vaccines [152]. Research teams have developed circulating tumor DNA (ctDNA)-based biomarkers to monitor tumors directly through the detection of real-time evolution and therapy response, as well as resistance mechanisms. Researchers have developed technological updates that will give clinicians access to adaptable treatment plans for the future [144].

#### 2.7.4 Novel immunotherapeutic approaches

Scientists are developing engineered synthetic high-density lipoproteins (sHDL) for personalized immunotherapy applications that act as nanoparticle-based immune stimulators to regulate tumor-associated macrophages and DCs [153]. Immune-engineered biomaterials represent a major advancement through their function as artificial lymph nodes for better T-cell activation, along with enhanced antigen presentation. The bioengineered implants offer extended immune activation against tumors, so they improve how well immunotherapy works over long periods of time [154]. Tumor-infiltrating lymphocyte therapy represents a new development as medical professionals expand T-cells derived from patients to reintroduce them into the body. Scientific studies have demonstrated that this therapy produces significant anti-tumor outcomes among patients whose solid tumors resist treatment [155].

### 2.8 Clinical translation: From preclinical models to clinical trials

Several therapies have translational issues despite promising findings in preclinical discussion of nanocarrier-based delivery and immune-enhancing biomaterials. The encouraging evidence of human efficacy is provided by recent clinical trial programs, e.g., KEYNOTE-942, that showed an improvement in survival in melanoma using mRNA-based neoantigen vaccines. In this Phase II trial, combination therapy with personalized vaccines and pembrolizumab showed a significant increase in recurrence-free survival over monotherapy, indicating how early leadership in preclinical understanding is starting to translate into clinical gains [156]. Other trials, mentioned in Table 5 have also moved beyond early-phase safety studies, suggesting a maturing field ripe for broader clinical adoption.

**Table 5:** Summary of clinical trials evaluating immunotherapeutic strategies

Study type	Cancer type	Immunotherapy agent	Immunotherapy strategy	Patient population	Key findings	References
Phase III (CheckMate 141 - TBP Subanalysis)	HNSCC	Nivolumab (PD-1)	Treatment Beyond Progression (TBP)	Post-platinum, RECIST-progressed patients allowed TBP with stable performance	25% had stable tumor burden; 25% had tumor reduction; overall survival (OS) = 12.7 months; no new safety signals	[157]
Phase III (OAK Trial - TBP subanalysis)	NSCLC	Atezolizumab (PD-L1)	TBP allowed post-progression	Patients continuing atezolizumab after radiographic progression	Post-progression OS = 12.7 months (vs 2.2 months no further tx); 49% stable lesions; no increased toxicity	[158]
Phase II study	NSCLC (Stage I–IV, post-surgery)	Autologous CIK cells	Adoptive cell therapy + chemotherapy	87 matched patients per arm (chemo vs chemo + CIK)	Advanced-stage OS = 24 vs 10 months (*p* < 0.001); early-stage 3-year OS = 82 vs 66%; progression free survival (PFS) and OS improved with more CIK cycles	[159]
Phase I/II	Prostate cancer	Peptide-pulsed DCs (PSM-P1, PSM-P2)	Vaccine-based adoptive therapy	Immunocompetent patients assessed for cytokine response	Response correlated with skin antigen reactivity and T-cell cytokine production; potential biomarker for inclusion	[160]
Pooled, retrospective analysis from Phase III trials (CheckMate 066 & 067)	Advanced Melanoma	Nivolumab (PD-1)	TBP	Treatment-naive patients from CheckMate 066 and 067; 85 patients received nivolumab >6 weeks after RECIST-defined progression	28% of TBP group had >30% tumor reduction after progression; 76% still alive at analysis; grade 3–4 adverse events similar to non-TBP group	[161]
Phase II, open-label, single-arm (KEYNOTE-427, Cohort A)	Advanced clear cell renal cell carcinoma (ccRCC)	Pembrolizumab (PD-1)	Monotherapy, first-line	110 treatment-naive patients with advanced ccRCC	ORR = 36.4% (3.6% CR, 32.7% PR); 58.2% disease control rate; median progression-free survival (PFS) = 7.1 months; 12- and 24-month OS = 88.2 and 70.8%; durable responses in all risk groups; 30% experienced grade 3–5 adverse events (colitis, diarrhea most common)	[162]
Phase I/IIa, dose-escalation	NSCLC	CV9201 (RNActive^®^ mRNA vaccine)	Multi-antigen mRNA vaccine targeting 5 NSCLC-associated antigens	46 patients with locally advanced (*n* = 7) or metastatic (*n* = 39) NSCLC post–first-line therapy, all with ≥ stable disease	Well-tolerated at all doses; 63% had antigen-specific immune responses; 60% had ≥ 2× increase in activated IgD⁺CD38^hi^ B cells; median PFS = 5.0 month, OS = 10.8 month; 2- and 3-year survival = 26.7 and 20.7%; no related grade 4/5 adverse events (AEs)	[163]
Phase Ib	Stage IV NSCLC	BI1361849 (CV9202)	mRNA vaccine (6 antigens) + local radiation ± maintenance therapy	26 patients (stratified by histology and EGFR mutation status) with PR/SD after first-line therapy	84% showed increased antigen-specific immune responses; 80% had higher antibody levels, 40% functional T-cell increases; SD in 46.2%, PR in 1 pt; vaccine well tolerated with mostly mild/moderate flu-like symptoms; supports further combination studies	[164]
Phase I (KEYNOTE**-**603)	Resected NSCLC (Part A) and Resected Melanoma (Part D)	mRNA-4157 (V940) ± Pembrolizumab	Individualized neoantigen mRNA vaccine, with/without checkpoint inhibitor	4 NSCLC patients (mRNA-4157 alone), 12 melanoma patients (mRNA-4157 + pembrolizumab)	Well tolerated; no grade 4/5 AEs; mRNA-4157 induced robust *de novo* and boosted preexisting T-cell responses; combination with pembrolizumab expanded cytotoxic CD4⁺ and CD8⁺ T cells	[165]
Phase I/II (First-in-human, open-label)	Multiple solid tumors (melanoma, NSCLC, bladder, HNSCC, MSS-CRC, BCC, TNBC)	mRNA-4359 ± Pembrolizumab	Lipid nanoparticle mRNA vaccine encoding PD-L1 and IDO1 peptides; monotherapy and combo with PD-1 inhibitor	Dose escalation (Arm 1a): refractory advanced/metastatic tumors; Combo (Arm 1b/Arm 2): ICI-refractory or ICI-naive melanoma and NSCLC	Designed to activate PD-L1/IDO1-specific T-cells to reverse immunosuppression; aims to kill both tumor and regulatory immune cells	[166]

### 2.9 AI in immunotherapy

The future of personalized medicine in immunotherapy emphasizes biomarker-driven approaches, innovative modalities, and AI integration to enhance cancer treatment outcomes (Table 6). As part of CIT strategies, AI has progressively become a key component, potentially providing transformational methods of enhancing treatment precision, outcome prediction, and personalized treatment. AI algorithms, notably machine learning, have been applied to define biomarkers that show treatment effectiveness, such as PD-L1 expression levels and Tumor Mutation Burden. These biomarkers assist in determining the chances of a patient’s response towards immunotherapy. Works referring to Xie et al. reveal how imaging omics can be used to investigate tumor heterogeneity with regard to immunotherapy response and biomarker expression [167]. AI plays an important role in differentiating patients about their responsiveness to immunotherapy using large datasets such as genomics and imaging data. This improves the outcomes of treatment and minimizes the patient’s exposure to the wrong treatment plan [168]. It has been anticipated that neoantigens play a vital role in devising a cancer vaccine because of recognizing tumor-specific antigens. AI helps in the construction of these vaccines because it can predict the neoantigen presentation and immunogenicity [169]. By contrast, AI tools adapt tool strategies based on the response of the patient to interventions using longitudinal data. Integrated imaging biomarkers improve upon conventional evaluations, as described by Ghaffari Laleh et al., by providing ongoing feedback [170]. AI facilitates personalizing immunotherapy intervention based on multi-omics analysis tailored to the individual tumor. AI’s cooperation with omics technologies increases the speed of finding therapeutic targets [171].

**Table 6:** Future directions in personalized medicine

Focus area	Description	Examples/insights	Challenges	Future impact	Technologies involved	References
Biomarker development	Identification of predictive biomarkers for therapy optimization	Genomic technologies, AI to discover robust biomarkers for immunotherapies	Variability in patient genetic profiles, high costs of validation	Improved treatment precision and reduced adverse effects	Genomic sequencing, AI algorithms	[172,173]
New immunotherapy modalities	Emerging treatments targeting specific antigens or immune pathways	Neoantigen-based vaccines, bispecific antibodies, engineered TCR therapies	Limited efficacy in heterogeneous tumors, logistical complexities	Expanded therapeutic arsenal, tackling resistant cancers	Synthetic biology, CRISPR, mRNA technology	[174]
AI & big data integration	Use of AI to analyze complex datasets for therapy customization	AI predicts treatment responses, tumor evolution, and optimal therapy combinations	Data privacy concerns, integration with clinical workflows	Accelerated discovery of treatment pathways and improved patient stratification	Machine learning, Natural Language Processing (NLP)	[175]
Combination therapy	Integration of immunotherapy with other treatments	Synergistic effects with targeted therapy, chemotherapy, or radiation	Complex clinical trial designs, managing cumulative side effects	Enhanced efficacy and reduced therapeutic resistance	Multimodal therapy platforms, computational biology	[176,177]
TME	Modulating the tumor’s immune environment to improve therapy outcomes	Targeting suppressive immune cells and reprogramming the immune landscape	Incomplete understanding of microenvironment dynamics	Boosted immune system response and minimized immune evasion by tumors	Cellular therapy, immune modulators, high-resolution imaging	[178]
Focus on hard-to-treat cancers	Novel approaches for cancers resistant to current treatments	DCs vaccines, personalized peptide vaccines	High costs, lack of universal efficacy, and limited clinical data	Targeted treatment options for previously untreatable cancers	Vaccine development, peptide synthesis, proteomics	[179]
Accessibility and equity	Ensuring global access to personalized immunotherapy treatments	Reduced costs, simplified diagnostics, and equitable therapies	High cost of therapies, and lack of infrastructure in low-income regions	Wider adoption of personalized treatments, reduced global health disparities	Point-of-care diagnostics, low-cost platforms.IR	[180]

AI-powered platforms such as DeepNeo, pVACtools, and NetMHCpan are revolutionizing neoantigen prediction and vaccine design by integrating multi-omics data for epitope identification (Table 7). However, clinical adoption is challenged by issues like algorithm transparency, regulatory validation, and a lack of interpretability in real-time decision-making [181].

**Table 7:** Summary of some neoantigen prediction tools

Tool	Methodology	Interpretability	Clinical integration	Key challenges	References
DeepNeo/DeepNeo-v2	Deep learning–based peptide–MHC binding and immunogenicity predictor	Moderate – black-box models; enhanced in v2 via improved rank metrics	Web server available; widely used for preclinical vaccine design	Limited transparency; needs clinical validation	[182]
DeepNeo-AGNet	Graph attention network + CNN for capturing neoantigen patterns	Moderate – architecture complexity limits user explainability	Early-stage; research-focused	Deep model complexity and reproducibility	[183]
ImmuScope	Self-iterative multiple-instance learning for CD4⁺ T-cell epitope prediction	High – allows feature attribution and region activation analysis	Integrates with antigen presentation prediction; benchmarked against DeepNeo	Still under validation; heavy computational demand	[184]
HLAIImaster	Adaptive deep learning for HLA class II binding prediction	High – trained on domain-informed architectures	Focus on class II neoepitopes; useful for vaccine design	Limited support for rare HLA alleles	[185]
FusionHLAII	BiGRU + Transformer using physicochemical encoding	High – captures temporal and global interactions; interpretable attention	Open-source; early research stage	Requires benchmark validation and wider testing	[186]
THLANet	ESM-2 + Transformer-Encoder for TCR–neoantigen–HLA class I prediction	High – 3D binding site visualization and alanine scanning enhance explainability	Validated across cancer datasets; outperforms PanPep and pMTnet	TCR–pHLA binding is data-limited; early in clinical uptake	[187]

The development of personalized medicine and immunotherapy has greatly impacted the approaches to treating cancer by specifically targeting genetic and molecular markers. Colorectal cancer treatment has embraced precision medicine together with immunotherapy, involving genomic knowledge for targeted treatment [188]. Research stresses immunotherapy as a viable approach to ovarian cancer, especially for its inclusion in the future treatment plan of personalized medicine [189]. A new horizon for immunotherapy has emerged with gene editing and RNA interference technology in the treatment of patients.

### 2.10 Challenges in CIT

The clinical uptake of next-generation immunotherapies faces several systemic barriers. One of the most pressing barriers is the manufacturing complexity, particularly for personalized therapies like CAR T-cells and individualized neoantigen vaccines. CAR T therapy involves *ex vivo* genetic modification of a patient’s own T-cells, a process that takes 2–3 weeks and requires highly controlled good manufacturing practice environments. This time lag is problematic in aggressive cancers where rapid progression outpaces therapy preparation. Conversely, neoantigen vaccines, such as mRNA-4157 and CV9202, continue to mandate labor-intensive antigen identification, peptide synthesis, and formulations with turnaround times extending to more than 1 month, and thus are non-viable in clinical settings [182]. AI tools themselves, as computation pipeline technologies accelerate prediction, even though the framework requires timely tumor exome sequencing and HLA typings that are not equally accessible in healthcare environments. Workflows are also often delayed by data bottlenecks and inconsistency of sample quality, which do not allow their timely integration with the therapeutic decision-making [190].

Regulatory pathways remain unclear for many neoantigen-based therapies. Whereas ICIs and CAR T-cells have made it through regulatory approval with standardized clinical measures (e.g., ORR, PFS), neoantigen vaccines and AI-predicted epitope treatments have no normalized surrogate measures and population-wide clinical trial design. In principle, phase III designs are not easily affected by personalized vaccine trials owing to the lack of sample size and standardization. Regulatory agencies like the U.S. Food and drug administration and the European Medicines Agency require both regulation and scalability, which are both overstretched in high-personalized mutanome and immune profiling-driven therapies. Moreover, there is also the problem of interpretability of AI-based tools, which establishes a bottleneck for clinical validation. These models often operate as “black boxes,” challenging traditional regulatory frameworks that require explainable outputs, especially when integrated into diagnostics or companion decision tools [191]. The economic burden of immunotherapy remains one of the most well-documented obstacles to widespread use.

Global disparities in immunotherapy access remain stark. Access to CAR T and ICIs is mostly available in high-income markets, whereas limited infrastructure and funding exist in low- and middle-income countries to conduct clinical trials and provide delivery systems. Furthermore, racial and ethnic minorities are not well-represented in clinical trials, distorting efficacy and toxicity data. To bridge this gap, strategies such as global licensing, public-private manufacturing partnerships, and regional regulatory harmonization are essential.

## 3 Conclusion

Advances in personalized medicine, particularly in the realm of CIT, signify a transformative era in oncology. Immunotherapy has brought innovative strategies for cancer treatment by stimulating the human immune system to resist tumors. While it demonstrates promising application effects, it is not without certain defects and limitations. This study analyzed recent advances and the current status of immunotherapy research, emphasizing emerging tools such as nanomedicine formulations that provide targeted cancer therapies. The use of T-cell subsets, novel antigen expression methods, and the development of interventions targeting the TME hold significant potential. Neoadjuvant ICI blockades and combined immunotherapeutic approaches further enhance anti-tumor efficacy. Additionally, the integration of AI into CIT marks a critical leap forward. Innovations in deep learning, NLP, and federated learning are expected to expand the scope of biomarker discovery and predictive modeling. AI’s role in clinical workflows will likely accelerate personalized therapeutic decisions while reducing the time and costs associated with drug development and clinical trials.

Future research must prioritize individualized detection and management, the rapid development of new technologies and vaccines targeting novel antigens, the reduction of side effects, and the mitigation of immunotherapy-associated drug resistance. Looking ahead, the convergence of personalized medicine, immunotherapy, and AI offers a transformative potential to revolutionize cancer treatment. Through continued innovation and efforts to address existing challenges, this multidisciplinary approach promises not only improved patient outcomes but also a future where cancer care is highly efficient, targeted, and accessible for all.
